# Supplementary material for: Identifying accurate metagenome and amplicon software via a meta-analysis of sequence to taxonomy benchmarking studies
Source: PeerJ. 2019 Jan 4;7:e6160. doi: 10.7717/peerj.6160 (PMC6322486; doi:10.7717/peerj.6160)
Supplement: Supplemental Information 1 [file peerj-07-6160-s001.pdf]

Supplementary results for: Identifying accurate metagenome and amplicon software via a meta-analysis of sequence to taxonomy benchmarking studies

Paul P. Gardner[1,2], Renee J. Watson[1], Xochitl C. Morgan[3], Jenny L. Draper[4], Robert D. Finn[5], Sergio E. Morales[3], and Matthew B. Stott[1]

[1]**Biomolecular Interaction Centre, School of Biological Sciences, University of Canterbury, Christchurch, New Zealand.**
[2]**Department of Biochemistry, University of Otago, Dunedin, New Zealand.**
[3]**Department of Microbiology and Immunology, University of Otago, Dunedin, New Zealand.**
[4]**Institute of Environmental Science and Research, Porirua, New Zealand.**
[5]**European Molecular Biology Laboratory, European Bioinformatics Institute (EMBL-EBI), Wellcome Trust Genome Campus, Hinxton, Cambridge CB10 1SD, UK**

Corresponding author:

Paul P. Gardner[1]

Email address: paul.gardner@otago.ac.nz

ABSTRACT

In the below we provide additional results for our investigation into benchmarks of metagenomics analysis tools.

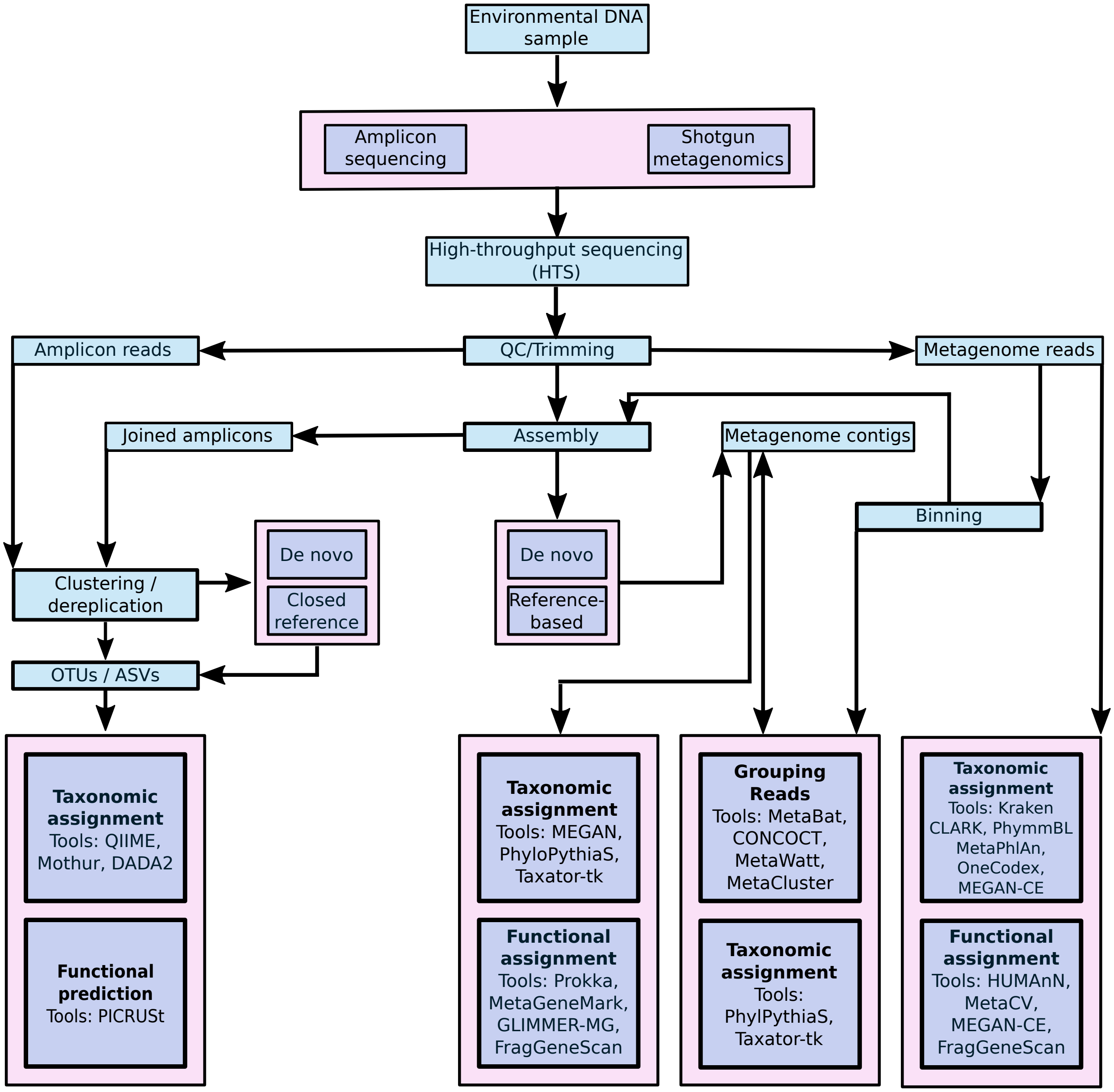

Figure S1. A high-level summary of the main metagenome data production and analysis pathways. The main split is between amplicon or marker-gene based approaches and the shotgun metagenomics strategies. These sequences can be further processed and used to generate Operational Taxonomic Units (OTUs), Amplicon Sequence Variants (ASVs), and/or mapped onto reference databases for taxonomic and/or functional assignments. The tools referenced in the figure include QIIME (Caporaso et al., 2010), Mothur (Schloss et al., 2009), DADA2 (Callahan et al., 2016), PICRUSt (Langille et al., 2013), MEGAN (Huson et al., 2007), PhyloPythiaS (Gregor et al., 2016), Taxator-tk (Dröge et al., 2015), Prokka (Seemann, 2014), MetaGeneMark (Zhu et al., 2010), GLIMMER-MG (Kelley et al., 2012), FragGeneScan (Rho et al., 2010), Kraken (Wood and Salzberg, 2014), CLARK (Ounit et al., 2015), PhymmBL (Brady and Salzberg, 2011), MetaPhlAn (Truong et al., 2015), One Codex (Minot et al., 2015), MEGAN-CE (Huson et al., 2016), HUMAnN (Abubucker et al., 2012) and MEtaCV (Liu et al., 2013).

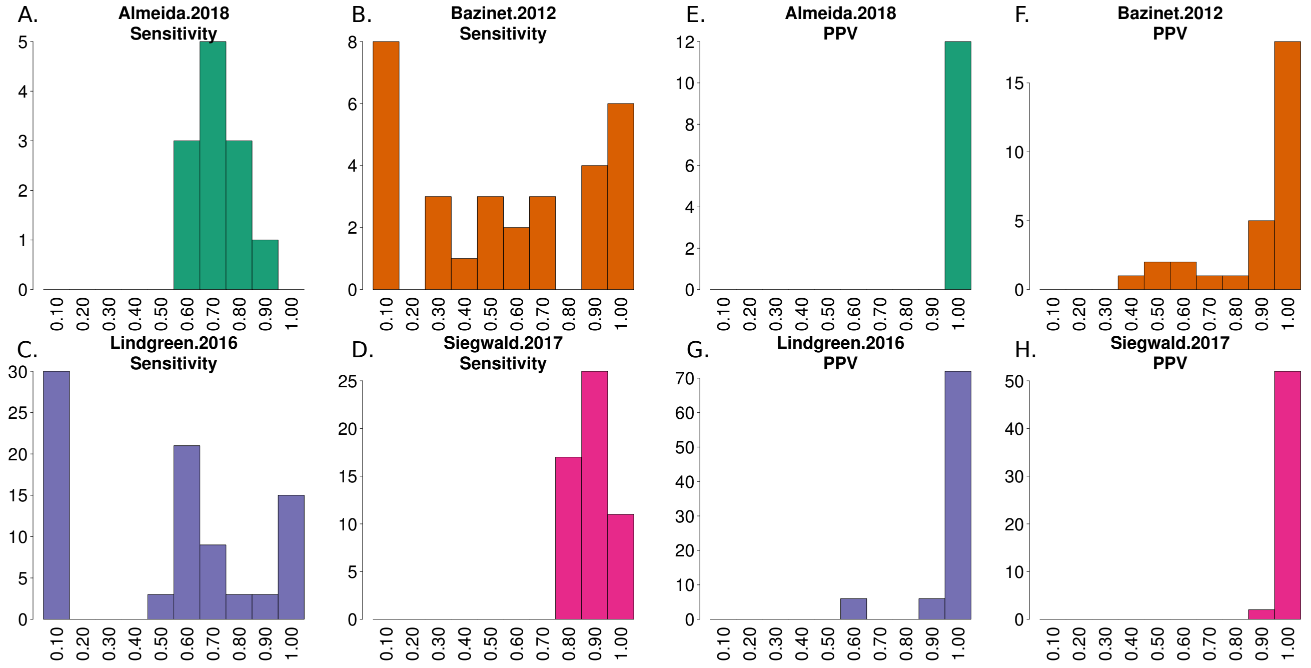

Figure S2. The distribution of sensitivity (A-D) and PPV (E-H) estimates for each of the four benchmark publications. Each benchmark has a different characteristic distribution for sensitivity and PPV due to different test dataset sizes and different methods for computing these values.

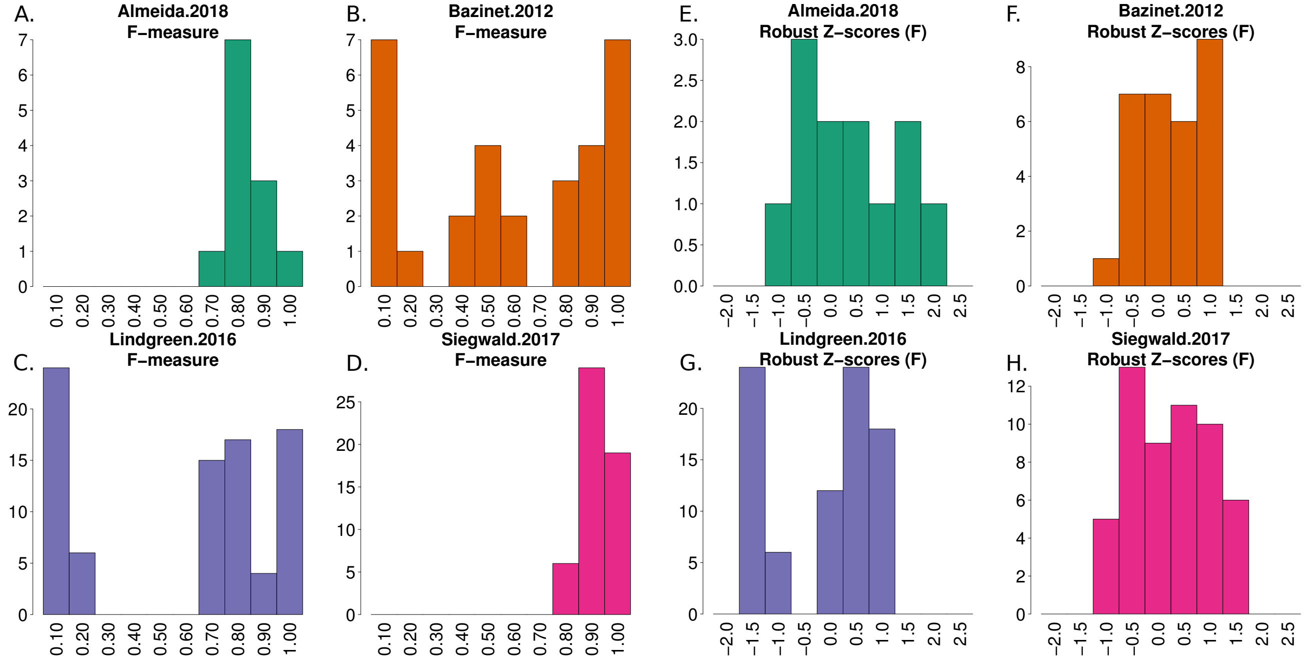

Figure S3. The distributions of F-measure estimates and corresponding robust Z-scores for each of the six benchmark publications. Each benchmark has a different characteristic distribution for F-measure due to different test dataset sizes and different methods for computing F-measure. The robust Z-score corrects for some of the variation between benchmarks.

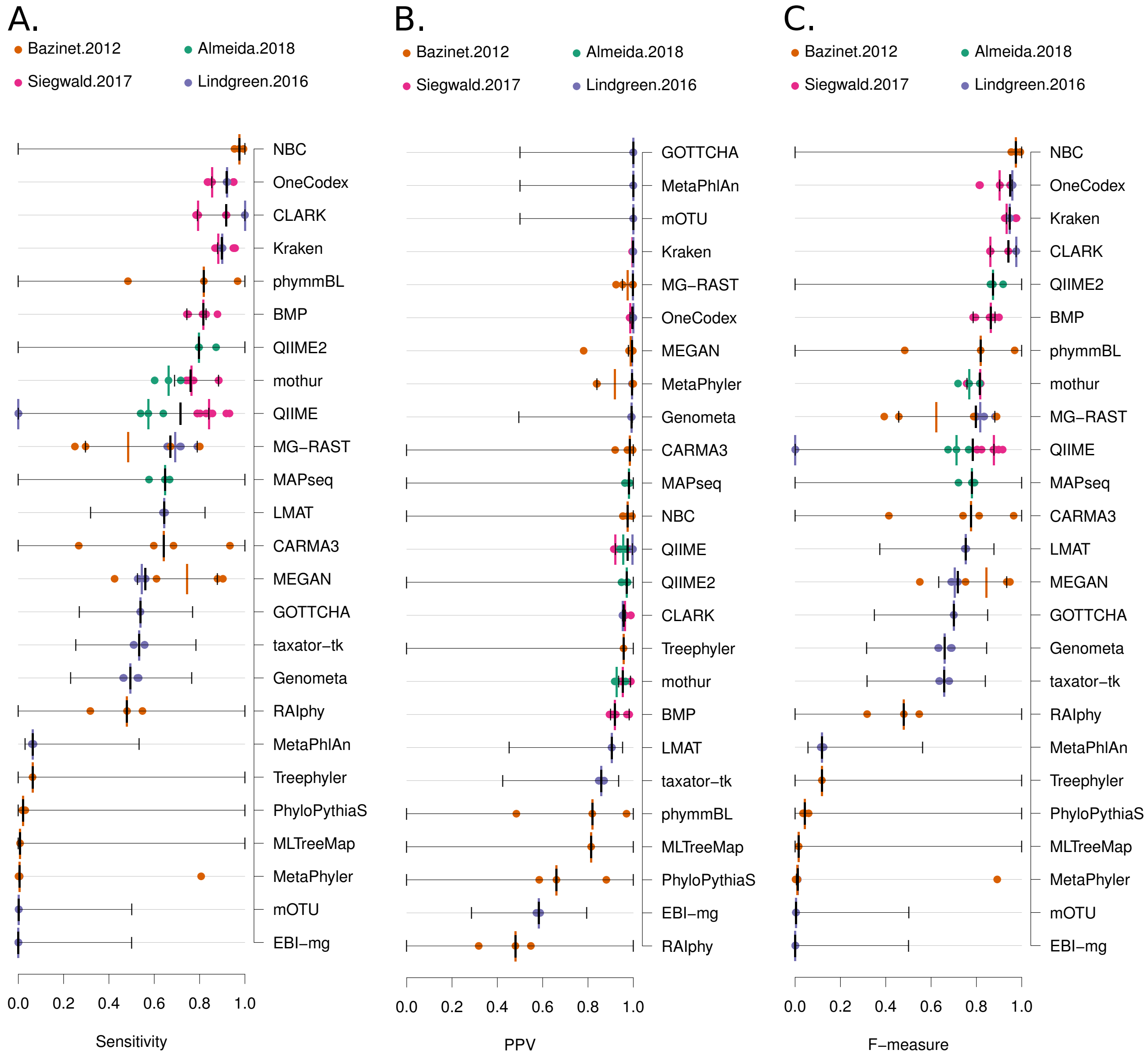

Figure S4. Ranked lists of metagenome analysis tools, based upon median Sensitivity, PPV and F measures. Coloured points indicate an estimated accuracy measure from one of four benchmark publications. Median values are indicated by a vertical bar (black for the overall median value, coloured bars for the median value from a publication). Bootstrap derived 95% confidence intervals for the Sensitivity, PPV or F-measure are indicated with a thin black lines for each method.

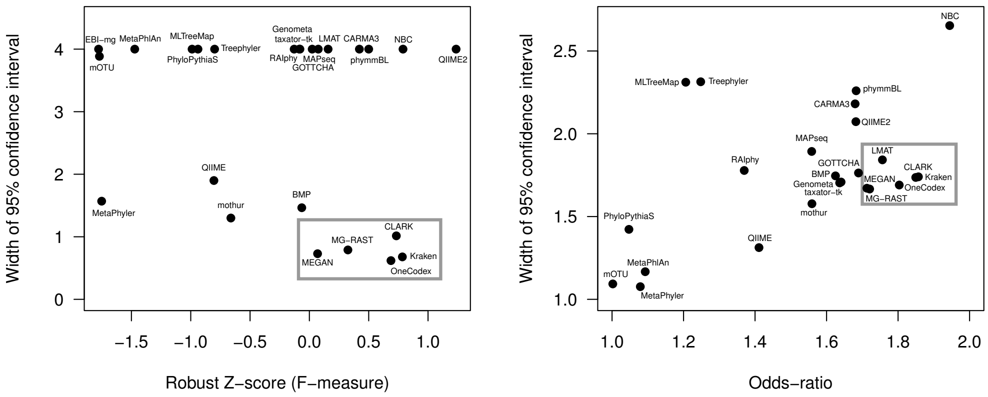

Figure S5. Estimated effect size (Robust Z-scores or Odds ratios) versus the confidence intervals. These plots show an alternative view of the forest-plots from Figure 3B and Figure 4A. The small sets of tools with comparatively high estimated accuracy and small confidence intervals have been indicated with grey boxes.

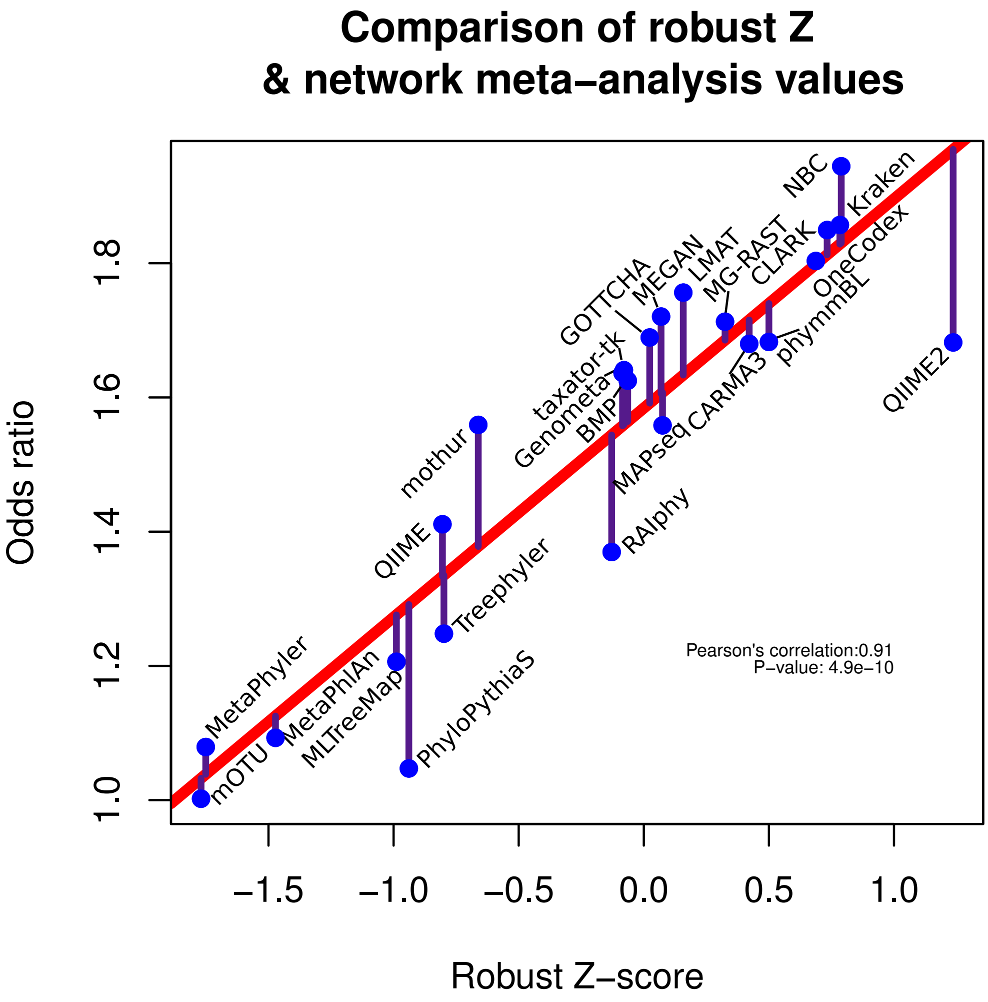

Figure S6. Comparison of Robust Z-scores and odds ratios from the network meta-analysis. The Pearson's correlation coefficient between the two approaches for ranking software tools is 0.91 (P-value=4.9×10^{-10}).

Paper	Principle 1: study focus is an evaluation	Principle 2: authors should be reasonably neutral	Principle 3: test data, evaluation and metrics should be rational
Almeida et al. (2018)	Yes	Yes	Yes
Bazinet et al. (2012)	Yes	Yes	Yes
Lindgreen et al. (2016)	Yes	Yes	Yes
McIntyre et al. (2017)	Yes	No*	Yes
Peabody et al. (2015)	Yes	Yes	No***
Sczyrba et al. (2017)	Yes	No**	Yes
Siegwald et al. (2017)	Yes	Yes	Yes

Table S1. Supplementary Table 1: There are three main criterias for benchmarking that authors should try to adhere to (Boulesteix et al., 2013). Criteria 1: the main focus of the study should be an evaluation. This criteria was evaluated manually be the authors of this study. Criteria 2: benchmark authors should be reasonably neutral i.e. not involved in the development of methods included in the evaluation. This was evaluated by collecting method references provided by benchmark authors, and were tabulated and evaluated manually for overlap between authorship lists for the benchmarks and methods. Criteria 3: the test data, evaluation and methods should be selected in a rational way. We assessed the number of taxa used and the evaluation metrics reported for each study. If either of these were too low or likely to be biased (e.g. only reporting sensitivity), then criteria 3 was not met.
*The benchmark co-authors S Lonardi and R Ounit are also co-authors of the tools CLARK and CLARK-S, GL Rosen is a co-author of the tool NBC. All three tools were benchmarked in this study.
**12 of the 67 CAMI benchmark authors are also co-authors for 7 of the 14 tools that were benchmarked in this study.
***Subsequent analysis of the results from this manuscript highlight that the 11 taxa used in this evaluation is too few for robust accuracy estimates. Furthermore, one of the taxa has been renamed in subsequent taxonomies, making some of the accuracy estimates lower than these are in practise (Lu et al., 2017).

REFERENCES

Abubucker, S., Segata, N., Goll, J., Schubert, A. M., Izard, J., Cantarel, B. L., Rodriguez-Mueller, B., Zucker, J., Thiagarajan, M., Henrissat, B., White, O., Kelley, S. T., Methé, B., Schloss, P. D., Gevers, D., Mitreva, M., and Huttenhower, C. (2012). Metabolic reconstruction for metagenomic data and its application to the human microbiome. *PLoS Comput. Biol.*, 8(6):e1002358.

Boulesteix, A.-L., Lauer, S., and Eugster, M. J. A. (2013). A plea for neutral comparison studies in computational sciences. *PLoS One*, 8(4):e61562.

Brady, A. and Salzberg, S. (2011). PhymmBL expanded: confidence scores, custom databases, parallelization and more. *Nat. Methods*, 8(5):367.

Callahan, B. J., McMurdie, P. J., Rosen, M. J., Han, A. W., Johnson, A. J. A., and Holmes, S. P. (2016). DADA2: High-resolution sample inference from illumina amplicon data. *Nat. Methods*, 13(7):581–583.

Caporaso, J. G., Kuczynski, J., Stombaugh, J., Bittinger, K., Bushman, F. D., Costello, E. K., Fierer, N., Peña, A. G., Goodrich, J. K., Gordon, J. I., Huttley, G. A., Kelley, S. T., Knights, D., Koenig, J. E., Ley, R. E., Lozupone, C. A., McDonald, D., Muegge, B. D., Pirrung, M., Reeder, J., Sevinsky, J. R., Turnbaugh, P. J., Walters, W. A., Widmann, J., Yatsunenko, T., Zaneveld, J., and Knight, R. (2010). QIIME allows analysis of high-throughput community sequencing data. *Nat. Methods*, 7(5):335–336.

Dröge, J., Gregor, I., and McHardy, A. C. (2015). Taxator-tk: precise taxonomic assignment of metagenomes by fast approximation of evolutionary neighborhoods. *Bioinformatics*, 31(6):817–824.

Gregor, I., Dröge, J., Schirmer, M., Quince, C., and McHardy, A. C. (2016). PhyloPythiaS+: a self-training method for the rapid reconstruction of low-ranking taxonomic bins from metagenomes. *PeerJ*, 4:e1603.

Huson, D. H., Auch, A. F., Qi, J., and Schuster, S. C. (2007). MEGAN analysis of metagenomic data. *Genome Res.*, 17(3):377–386.

Huson, D. H., Beier, S., Flade, I., Górska, A., El-Hadidi, M., Mitra, S., Ruscheweyh, H.-J., and Tappu, R. (2016). MEGAN community edition - interactive exploration and analysis of Large-Scale microbiome sequencing data. *PLoS Comput. Biol.*, 12(6):e1004957.

Kelley, D. R., Liu, B., Delcher, A. L., Pop, M., and Salzberg, S. L. (2012). Gene prediction with glimmer for metagenomic sequences augmented by classification and clustering. *Nucleic Acids Res.*, 40(1):e9.

Langille, M. G. I., Zaneveld, J., Caporaso, J. G., McDonald, D., Knights, D., Reyes, J. A., Clemente, J. C., Burkepile, D. E., Vega Thurber, R. L., Knight, R., Beiko, R. G., and Huttenhower, C. (2013). Predictive functional profiling of microbial communities using 16S rRNA marker gene sequences. *Nat. Biotechnol.*, 31(9):814–821.

Liu, J., Wang, H., Yang, H., Zhang, Y., Wang, J., Zhao, F., and Qi, J. (2013). Composition-based classification of short metagenomic sequences elucidates the landscapes of taxonomic and functional enrichment of microorganisms. *Nucleic Acids Res.*, 41(1):e3.

Lu, J., Breitwieser, F. P., Thielen, P., and Salzberg, S. L. (2017). Bracken: estimating species abundance in metagenomics data. *PeerJ Comput. Sci.*, 3:e104.

Minot, S. S., Krumm, N., and Greenfield, N. B. (2015). One codex: a sensitive and accurate data platform for genomic microbial identification. *bioRxiv*.

Ounit, R., Wanamaker, S., Close, T. J., and Lonardi, S. (2015). CLARK: fast and accurate classification of metagenomic and genomic sequences using discriminative k-mers. *BMC Genomics*, 16:236.

Rho, M., Tang, H., and Ye, Y. (2010). FragGeneScan: predicting genes in short and error-prone reads. *Nucleic Acids Res.*, 38(20):e191.

Schloss, P. D., Westcott, S. L., Ryabin, T., Hall, J. R., Hartmann, M., Hollister, E. B., Lesniewski, R. A., Oakley, B. B., Parks, D. H., Robinson, C. J., Sahl, J. W., Stres, B., Thallinger, G. G., Van Horn, D. J., and Weber, C. F. (2009). Introducing mothur: open-source, platform-independent, community-supported software for describing and comparing microbial communities. *Appl. Environ. Microbiol.*, 75(23):7537–7541.

Seemann, T. (2014). Prokka: rapid prokaryotic genome annotation. *Bioinformatics*.

Truong, D. T., Franzosa, E. A., Tickle, T. L., Scholz, M., Weingart, G., Pasolli, E., Tett, A., Huttenhower, C., and Segata, N. (2015). MetaPhlAn2 for enhanced metagenomic taxonomic profiling. *Nat. Methods*, 12(10):902–903.

Wood, D. E. and Salzberg, S. L. (2014). Kraken: ultrafast metagenomic sequence classification using exact alignments. *Genome Biol.*, 15(3):R46.

Zhu, W., Lomsadze, A., and Borodovsky, M. (2010). Ab initio gene identification in metagenomic sequences. *Nucleic Acids Res.*, 38(12):e132.
